# Supplementary material for: Fermentation of cv. Kalamata Natural Black Olives with Potential Multifunctional Yeast Starters
Source: Foods. 2022 Oct 6;11(19):3106. doi: 10.3390/foods11193106 (PMC9563747; doi:10.3390/foods11193106)
Supplement: Supplementary file 1 [file foods-11-03106-s001.zip › foods-1931471-supplementary.pdf]

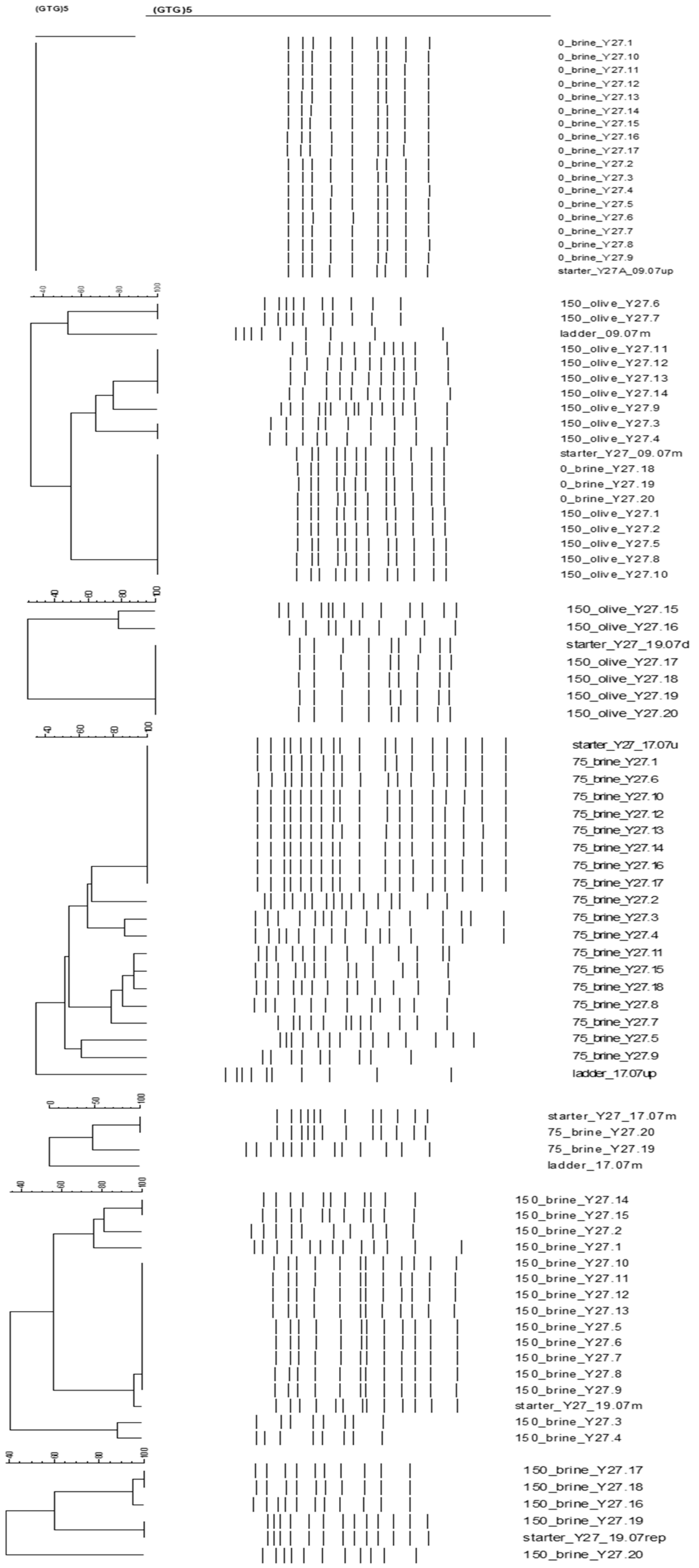
(GTG)5
(GTG)5
0_brine_Y27.1
0_brine_Y27.10
0_brine_Y27.11
0_brine_Y27.12
0_brine_Y27.13
0_brine_Y27.14
0_brine_Y27.15
0_brine_Y27.16
0_brine_Y27.17
0_brine_Y27.2
0_brine_Y27.3
0_brine_Y27.4
0_brine_Y27.5
0_brine_Y27.6
0_brine_Y27.7
0_brine_Y27.8
0_brine_Y27.9
starter_Y27A_09.07up
150_olive_Y27.6
150_olive_Y27.7
ladder_09.07m
150_olive_Y27.11
150_olive_Y27.12
150_olive_Y27.13
150_olive_Y27.14
150_olive_Y27.9
150_olive_Y27.3
150_olive_Y27.4
starter_Y27_09.07m
0_brine_Y27.18
0_brine_Y27.19
0_brine_Y27.20
150_olive_Y27.1
150_olive_Y27.2
150_olive_Y27.5
150_olive_Y27.8
150_olive_Y27.10
150_olive_Y27.15
150_olive_Y27.16
starter_Y27_19.07d
150_olive_Y27.17
150_olive_Y27.18
150_olive_Y27.19
150_olive_Y27.20
starter_Y27_17.07u
75_brine_Y27.1
75_brine_Y27.6
75_brine_Y27.10
75_brine_Y27.12
75_brine_Y27.13
75_brine_Y27.14
75_brine_Y27.16
75_brine_Y27.17
75_brine_Y27.2
75_brine_Y27.3
75_brine_Y27.4
75_brine_Y27.11
75_brine_Y27.15
75_brine_Y27.18
75_brine_Y27.8
75_brine_Y27.7
75_brine_Y27.5
75_brine_Y27.9
ladder_17.07up
starter_Y27_17.07m
75_brine_Y27.20
75_brine_Y27.19
ladder_17.07m
150_brine_Y27.14
150_brine_Y27.15
150_brine_Y27.2
150_brine_Y27.1
150_brine_Y27.10
150_brine_Y27.11
150_brine_Y27.12
150_brine_Y27.13
150_brine_Y27.5
150_brine_Y27.6
150_brine_Y27.7
150_brine_Y27.8
150_brine_Y27.9
starter_Y27_19.07m
150_brine_Y27.3
150_brine_Y27.4
150_brine_Y27.17
150_brine_Y27.18
150_brine_Y27.16
150_brine_Y27.19
starter_Y27_19.07rep
150_brine_Y27.20

(A)

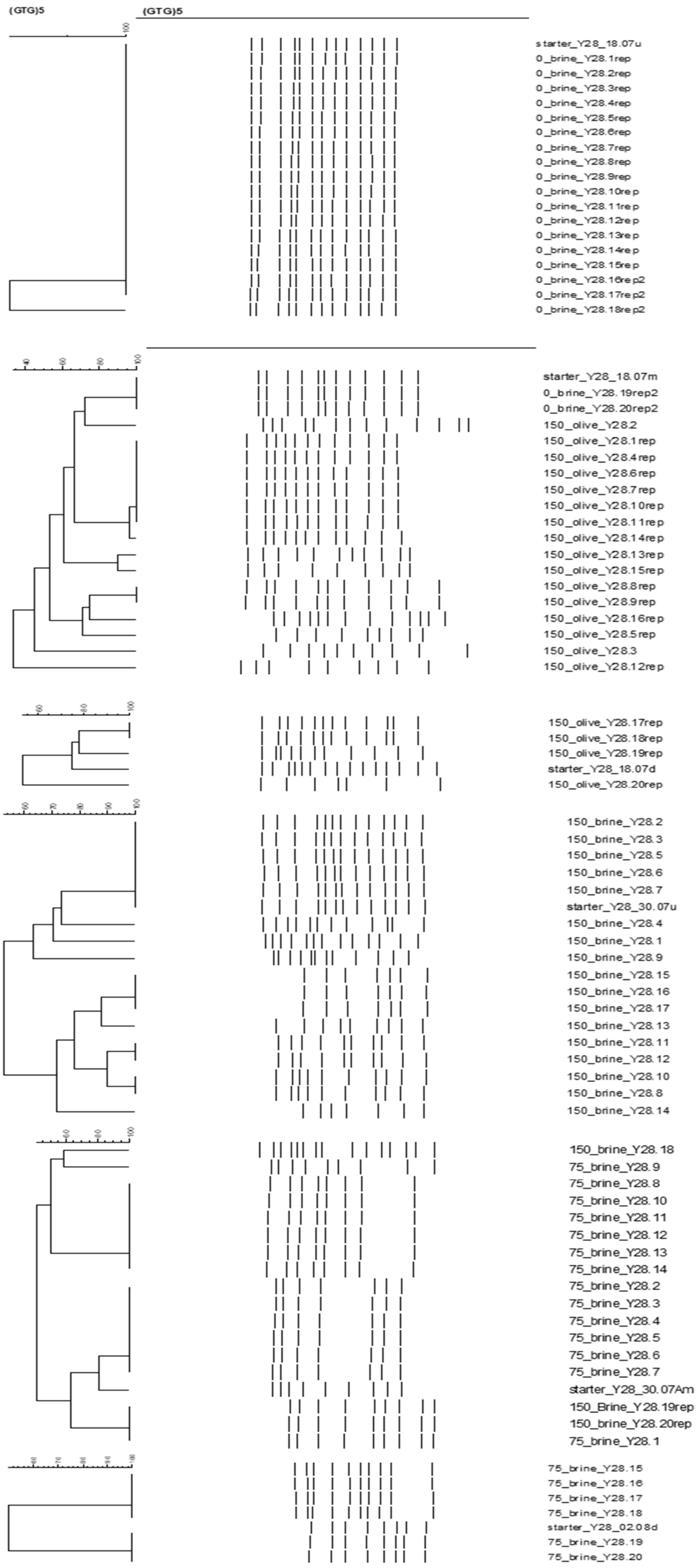
(GTG)5
(GTG)5
starter_Y28_18.07u
0_brine_Y28.1rep
0_brine_Y28.2rep
0_brine_Y28.3rep
0_brine_Y28.4rep
0_brine_Y28.5rep
0_brine_Y28.6rep
0_brine_Y28.7rep
0_brine_Y28.8rep
0_brine_Y28.9rep
0_brine_Y28.10rep
0_brine_Y28.11rep
0_brine_Y28.12rep
0_brine_Y28.13rep
0_brine_Y28.14rep
0_brine_Y28.15rep
0_brine_Y28.16rep2
0_brine_Y28.17rep2
0_brine_Y28.18rep2
starter_Y28_18.07m
0_brine_Y28.19rep2
0_brine_Y28.20rep2
150_olive_Y28.2
150_olive_Y28.1rep
150_olive_Y28.4rep
150_olive_Y28.6rep
150_olive_Y28.7rep
150_olive_Y28.10rep
150_olive_Y28.11rep
150_olive_Y28.14rep
150_olive_Y28.13rep
150_olive_Y28.15rep
150_olive_Y28.8rep
150_olive_Y28.9rep
150_olive_Y28.16rep
150_olive_Y28.5rep
150_olive_Y28.3
150_olive_Y28.12rep
150_olive_Y28.17rep
150_olive_Y28.18rep
150_olive_Y28.19rep
starter_Y28_18.07d
150_olive_Y28.20rep
150_brine_Y28.2
150_brine_Y28.3
150_brine_Y28.5
150_brine_Y28.6
150_brine_Y28.7
starter_Y28_30.07u
150_brine_Y28.4
150_brine_Y28.1
150_brine_Y28.9
150_brine_Y28.15
150_brine_Y28.16
150_brine_Y28.17
150_brine_Y28.13
150_brine_Y28.11
150_brine_Y28.12
150_brine_Y28.10
150_brine_Y28.8
150_brine_Y28.14
150_brine_Y28.18
75_brine_Y28.9
75_brine_Y28.8
75_brine_Y28.10
75_brine_Y28.11
75_brine_Y28.12
75_brine_Y28.13
75_brine_Y28.14
75_brine_Y28.2
75_brine_Y28.3
75_brine_Y28.4
75_brine_Y28.5
75_brine_Y28.6
75_brine_Y28.7
starter_Y28_30.07Am
150_Brine_Y28.19rep
150_brine_Y28.20rep
75_brine_Y28.1
75_brine_Y28.15
75_brine_Y28.16
75_brine_Y28.17
75_brine_Y28.18
starter_Y28_02.08d
75_brine_Y28.19
75_brine_Y28.20

(B)

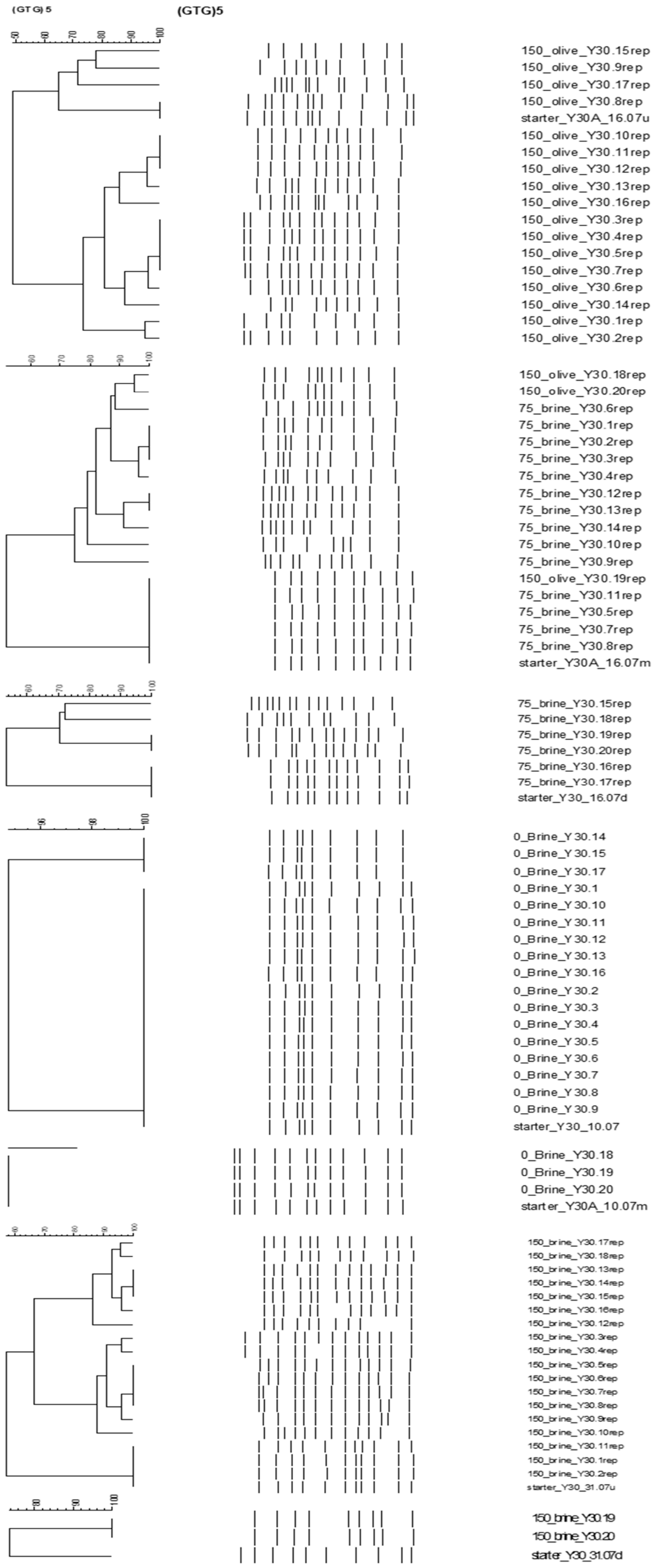

(GTG)5
(GTG)5
150_olive_Y30.15rep
150_olive_Y30.9rep
150_olive_Y30.17rep
150_olive_Y30.8rep
starter_Y30A_16.07u
150_olive_Y30.10rep
150_olive_Y30.11rep
150_olive_Y30.12rep
150_olive_Y30.13rep
150_olive_Y30.16rep
150_olive_Y30.3rep
150_olive_Y30.4rep
150_olive_Y30.5rep
150_olive_Y30.7rep
150_olive_Y30.6rep
150_olive_Y30.14rep
150_olive_Y30.1rep
150_olive_Y30.2rep
150_olive_Y30.18rep
150_olive_Y30.20rep
75_brine_Y30.6rep
75_brine_Y30.1rep
75_brine_Y30.2rep
75_brine_Y30.3rep
75_brine_Y30.4rep
75_brine_Y30.12rep
75_brine_Y30.13rep
75_brine_Y30.14rep
75_brine_Y30.10rep
75_brine_Y30.9rep
150_olive_Y30.19rep
75_brine_Y30.11rep
75_brine_Y30.5rep
75_brine_Y30.7rep
75_brine_Y30.8rep
starter_Y30A_16.07m
75_brine_Y30.15rep
75_brine_Y30.18rep
75_brine_Y30.19rep
75_brine_Y30.20rep
75_brine_Y30.16rep
75_brine_Y30.17rep
starter_Y30_16.07d
0_Brine_Y30.14
0_Brine_Y30.15
0_Brine_Y30.17
0_Brine_Y30.1
0_Brine_Y30.10
0_Brine_Y30.11
0_Brine_Y30.12
0_Brine_Y30.13
0_Brine_Y30.16
0_Brine_Y30.2
0_Brine_Y30.3
0_Brine_Y30.4
0_Brine_Y30.5
0_Brine_Y30.6
0_Brine_Y30.7
0_Brine_Y30.8
0_Brine_Y30.9
starter_Y30_10.07
0_Brine_Y30.18
0_Brine_Y30.19
0_Brine_Y30.20
starter_Y30A_10.07m
150_brine_Y30.17rep
150_brine_Y30.18rep
150_brine_Y30.13rep
150_brine_Y30.14rep
150_brine_Y30.15rep
150_brine_Y30.16rep
150_brine_Y30.12rep
150_brine_Y30.3rep
150_brine_Y30.4rep
150_brine_Y30.5rep
150_brine_Y30.6rep
150_brine_Y30.7rep
150_brine_Y30.8rep
150_brine_Y30.9rep
150_brine_Y30.10rep
150_brine_Y30.11rep
150_brine_Y30.1rep
150_brine_Y30.2rep
starter_Y30_31.07u
150_brine_Y30.19
150_brine_Y30.20
stater_Y30_31.07d

(C)

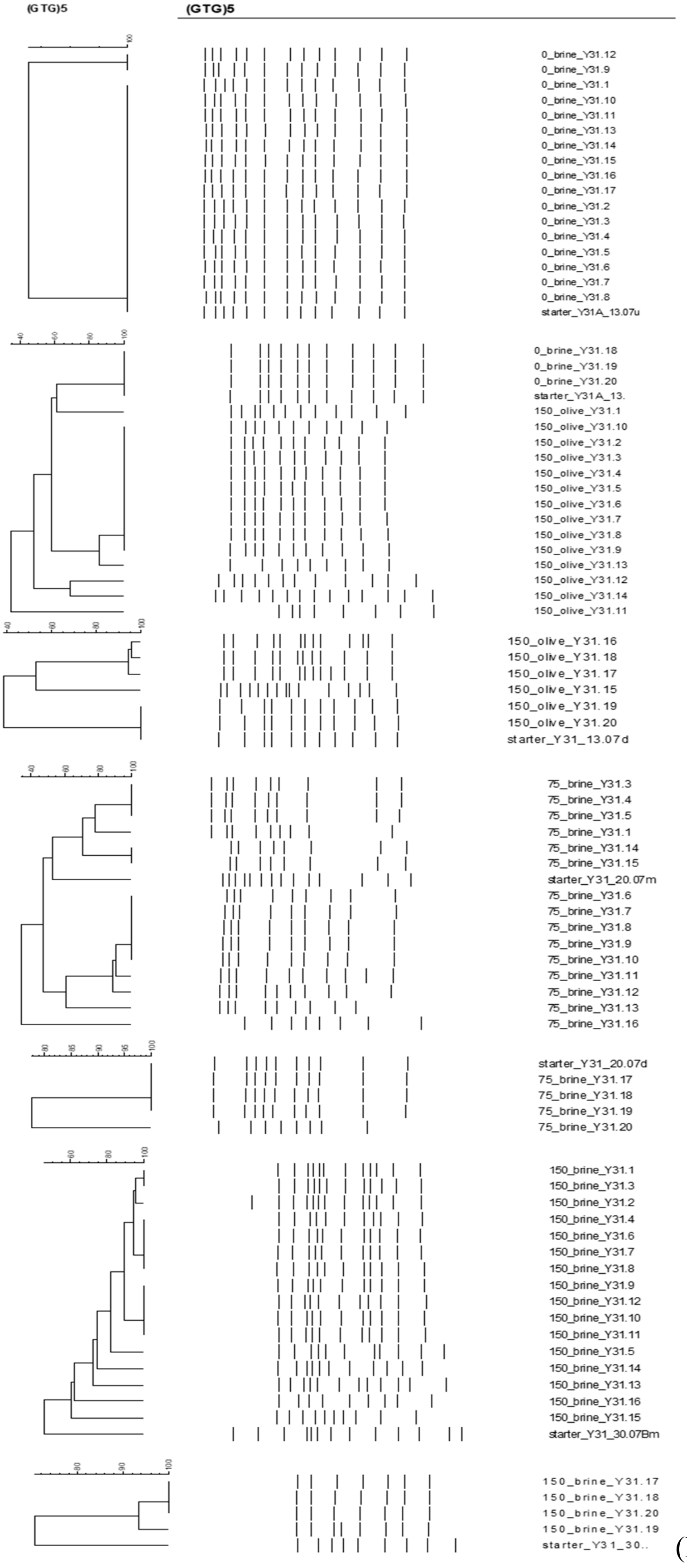
(GTG)5
(GTG)5
0_brine_Y31.12
0_brine_Y31.9
0_brine_Y31.1
0_brine_Y31.10
0_brine_Y31.11
0_brine_Y31.13
0_brine_Y31.14
0_brine_Y31.15
0_brine_Y31.16
0_brine_Y31.17
0_brine_Y31.2
0_brine_Y31.3
0_brine_Y31.4
0_brine_Y31.5
0_brine_Y31.6
0_brine_Y31.7
0_brine_Y31.8
starter_Y31A_13.07u
0_brine_Y31.18
0_brine_Y31.19
0_brine_Y31.20
starter_Y31A_13.
150_olive_Y31.1
150_olive_Y31.10
150_olive_Y31.2
150_olive_Y31.3
150_olive_Y31.4
150_olive_Y31.5
150_olive_Y31.6
150_olive_Y31.7
150_olive_Y31.8
150_olive_Y31.9
150_olive_Y31.13
150_olive_Y31.12
150_olive_Y31.14
150_olive_Y31.11
150_olive_Y31.16
150_olive_Y31.18
150_olive_Y31.17
150_olive_Y31.15
150_olive_Y31.19
150_olive_Y31.20
starter_Y31_13.07d
75_brine_Y31.3
75_brine_Y31.4
75_brine_Y31.5
75_brine_Y31.1
75_brine_Y31.14
75_brine_Y31.15
starter_Y31_20.07m
75_brine_Y31.6
75_brine_Y31.7
75_brine_Y31.8
75_brine_Y31.9
75_brine_Y31.10
75_brine_Y31.11
75_brine_Y31.12
75_brine_Y31.13
75_brine_Y31.16
starter_Y31_20.07d
75_brine_Y31.17
75_brine_Y31.18
75_brine_Y31.19
75_brine_Y31.20
150_brine_Y31.1
150_brine_Y31.3
150_brine_Y31.2
150_brine_Y31.4
150_brine_Y31.6
150_brine_Y31.7
150_brine_Y31.8
150_brine_Y31.9
150_brine_Y31.12
150_brine_Y31.10
150_brine_Y31.11
150_brine_Y31.5
150_brine_Y31.14
150_brine_Y31.13
150_brine_Y31.16
150_brine_Y31.15
starter_Y31_30.07Bm
150_brine_Y31.17
150_brine_Y31.18
150_brine_Y31.20
150_brine_Y31.19
starter_Y31_30..

(D)

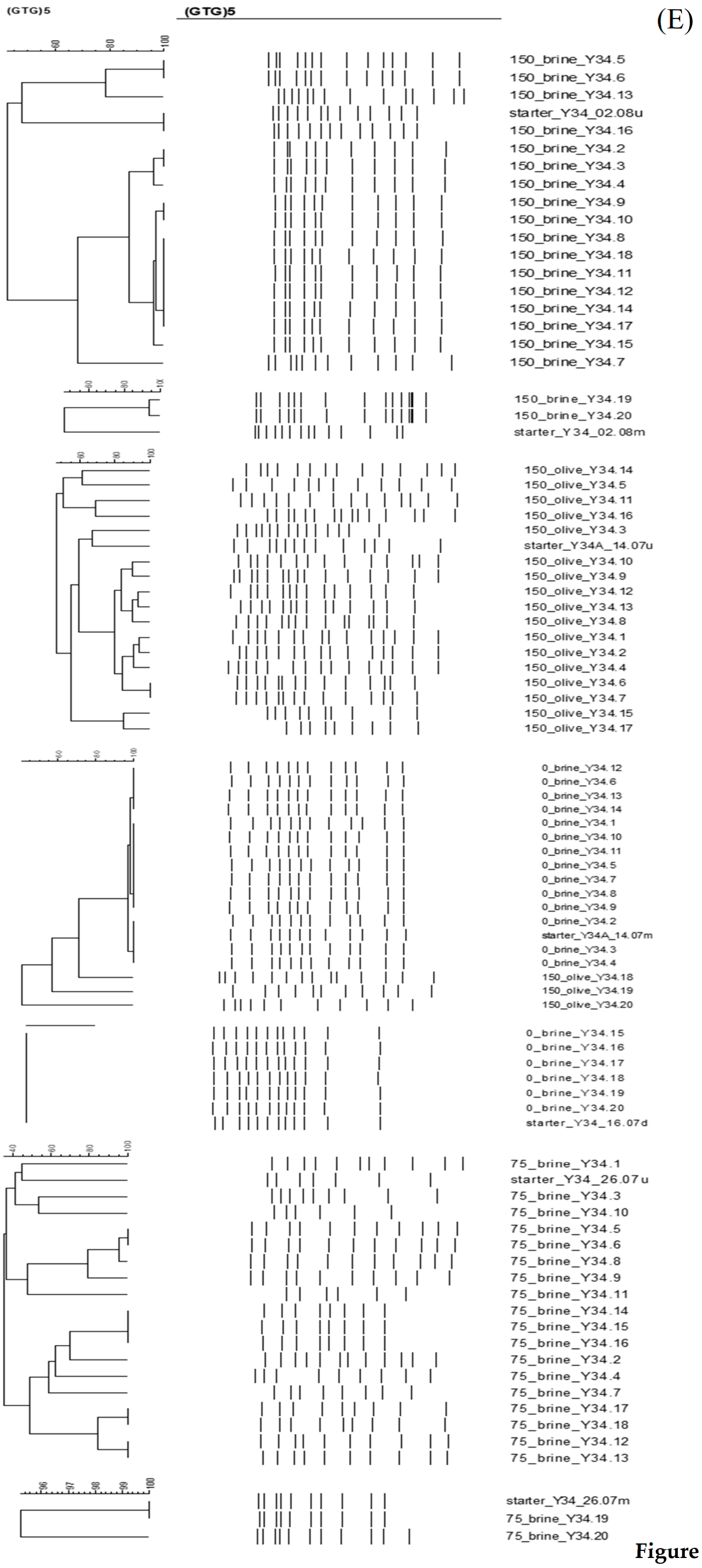

Figure S1. Cluster analysis of rep-PCR patterns of the isolates comparing to starters for the three sampling points (0, 75, and 150 days) in the brines and olives for the five inoculated fermentations: (A) *C. boidinii* Y27, (B) *C. boidinii* Y28, (C) *C. boidinii* Y30, (D) *C. boidinii* Y31, and (E) *S. cerevisiae* Y34. Distance is indicated by the mean correlation coefficient [r (%)] and clustering was performed by UPGMA analysis.
